# Proteomic differences in hippocampus and cortex of sudden unexplained death in childhood

**DOI:** 10.1007/s00401-022-02414-7

**Published:** 2022-03-25

**Authors:** Dominique F. Leitner, Christopher William, Arline Faustin, Manor Askenazi, Evgeny Kanshin, Matija Snuderl, Declan McGuone, Thomas Wisniewski, Beatrix Ueberheide, Laura Gould, Orrin Devinsky

**Affiliations:** 1 Comprehensive Epilepsy Center, Department of Neurology, NYU Langone Health and Grossman School of Medicine, New York, NY USA; 2 Department of Neurology, NYU Grossman School of Medicine, New York, NY USA; 3 Department of Pathology, NYU Grossman School of Medicine, New York, NY USA; 4 Center for Cognitive Neurology, NYU Grossman School of Medicine, New York, NY USA; 5 Biomedical Hosting LLC, Arlington, MA USA; 6 Proteomics Laboratory, Division of Advanced Research Technologies, NYU Grossman School of Medicine, New York, NY USA; 7 Department of Pathology, Yale School of Medicine, New Haven, CT USA; 8 Department of Biochemistry and Molecular Pharmacology, NYU Grossman School of Medicine, New York, NY USA; 9 Department of Psychiatry, NYU Grossman School of Medicine, New York, NY USA; 10 Sudden Unexplained Death in Childhood Foundation, New York, NY USA

**Keywords:** SUDC, Febrile seizures, Proteomics, Laser capture microdissection

## Abstract

Sudden unexplained death in childhood (SUDC) is death of a child over 1 year of age that is unexplained after review of clinical history, circumstances of death, and complete autopsy with ancillary testing. Multiple etiologies may cause SUDC. SUDC and sudden unexpected death in epilepsy (SUDEP) share clinical and pathological features, suggesting some similarities in mechanism of death and possible abnormalities in hippocampus and cortex. To identify molecular signaling pathways, we performed label-free quantitative mass spectrometry on microdissected frontal cortex, hippocampal dentate gyrus (DG), and cornu ammonis (CA1-3) in SUDC (*n* = 19) and pediatric control cases (*n* = 19) with an explained cause of death. At a 5% false discovery rate (FDR), we found differential expression of 660 proteins in frontal cortex, 170 in DG, and 57 in CA1-3. Pathway analysis of altered proteins identified top signaling pathways associated with activated oxidative phosphorylation (*p* = 6.3 × 10^–15^, *z* = 4.08) and inhibited EIF2 signaling (*p* = 2.0 × 10^–21^, *z* = − 2.56) in frontal cortex, and activated acute phase response in DG (*p* = 8.5 × 10^–6^, *z* = 2.65) and CA1-3 (*p* = 4.7 × 10^–6^, *z* = 2.00). Weighted gene correlation network analysis (WGCNA) of clinical history indicated that SUDC-positive post-mortem virology (*n* = 4/17) had the most significant module in each brain region, with the top most significant associated with decreased mRNA metabolic processes (*p* = 2.8 × 10^–5^) in frontal cortex. Additional modules were associated with clinical history, including fever within 24 h of death (top: increased mitochondrial fission in DG, *p* = 1.8 × 10^–3^) and febrile seizure history (top: decreased small molecule metabolic processes in frontal cortex, *p* = 8.8 × 10^–5^) in all brain regions, neuropathological hippocampal findings in the DG (top: decreased focal adhesion, *p* = 1.9 × 10^–3^). Overall, cortical and hippocampal protein changes were present in SUDC cases and some correlated with clinical features. Our studies support that proteomic studies of SUDC cohorts can advance our understanding of the pathogenesis of these tragedies and may inform the development of preventive strategies.

**Supplementary Information:**

The online version contains supplementary material available at 10.1007/s00401-022-02414-7.

## Introduction

Sudden unexplained death in childhood (SUDC) is death in a child (≥ 1 year of age) that remains unexplained despite review of clinical history, circumstances of death, and complete autopsy with ancillary testing [39]. Ancillary testing can include analysis of microbiology, toxicology, genetic, and metabolic testing [4, 59]. Different etiologies contribute to SUDC risk, including individual/familial febrile seizure history, undetected neuropathology, cardiac channelopathies, metabolic disorders, unidentified novel genetic variants, somatic mosaicism, and other factors and combinations thereof [27–29, 35, 47, 49, 52]. SUDC cases have a ten-fold higher prevalence of febrile seizure history than other children; their deaths typically occur during sleep, they are found prone, and are male [27, 28, 37, 49]. These circumstances are similar to sudden unexpected death in epilepsy (SUDEP) cases [1, 11, 28, 37]; thus, the mechanisms of death may overlap with SUDEP. Given the high prevalence of febrile seizures, the hippocampus is a region of interest in SUDC, especially since neuropathological changes similar to temporal lobe epilepsy (TLE) occur in many SUDC cases [26, 37, 38, 49]. However, controversy exists about these neuropathological changes as some investigators found many hippocampal findings within the range of normal variation [42, 48, 61].

There have been few molecular studies in SUDC and several related studies in sudden unexpected infant death (SUID) cases (< 1 year of age). Genetic variants may influence SUDC risk, particularly neurological and/or cardiomyopathies [8, 22, 24, 29, 52]. De novo mutations that occur post-zygotically can result in somatic mosaicism and occur in SUDC and SUDEP cases, which can influence brain development in the form of abnormal neuronal migration, brain overgrowth disorders, as well as epileptic encephalopathies, intellectual disability, autism, and neuropsychiatric diseases [10, 23]. To further evaluate molecular mechanisms, proteomic analyses have been performed in SUID and may provide implications for some SUDC cases [47]. SUID proteomics and protein analyses indicate differences in the 14-3-3 and serotonin signaling pathways, and potentially other pathways that require additional follow-up [3, 16, 17, 36]. There currently are no proteomic analyses reported in SUDC.

Our study identified differential expression of proteins in microdissected frontal cortex, hippocampal dentate gyrus (DG), and cornu ammonis (CA1-3) of SUDC cases and control cases with explained causes of death (COD). We evaluated autopsy formalin fixed paraffin embedded (FFPE) tissue to precisely microdissect brain regions from a section, the most readily available from archival autopsy material, and a robust source for proteomics analyses [12–15, 20, 30, 43, 60]. We identified the molecular signaling pathways associated with protein differences and how these protein changes correlated to SUDC clinical history and neuropathology.

## Materials and methods

### Human brain tissue

Post-mortem brain tissue from SUDC cases was obtained through the multisite collaboration with the SUDC Registry and Research Collaborative (SUDCRRC) with approval by the New York University School of Medicine Institutional Review Board (IRB #14-01061). The SUDCRRC evaluates cases from children ages 1 month to 18 years who have died suddenly and unexpectedly and the COD is unexplained after autopsy. Cases are referred from multiple sites, including NYU, Columbia University, the Mayo Clinic (Minnesota), and over 30 clinical and forensic collaborators at medical examiner and coroner offices. Ancillary analyses done at autopsy [8] included microbiology, with various testing performed (*n* = 17, all cases were not necessarily tested for all of the same viruses/bacteria). Of the cases with microbiology testing, 4/17 had positive post-mortem virology from respiratory and brain samples that were not considered as contributing to COD. Consent for SUDC cases was provided by the decedent’s parent(s)/guardian(s). After neuropathological review of the SUDC cases (CW, MS), brain tissue was processed into FFPE blocks. The neuropathology of the SUDC cases (17 of 19 cases) was previously described [49] and is detailed further in Supplementary Table 1, online resource. SUDC cases were included from those enrolled in the SUDCRRC from 2015 to 2018 with available brain tissue in the frontal cortex and hippocampus at the level of the lateral geniculate nucleus (LGN) [26, 37, 49]. SUDC cases were excluded (*n* = 3) when post-autopsy ancillary analyses identified potential pathogenic whole-exome sequencing (WES) variants that may contribute to COD [22]. Under IRB approval, de-identified control cases were obtained through partnerships with various medical examiner and coroner offices that were examined from 2012 to 2017 (*n* = 8). Additional control cases were obtained from the National Institutes of Health (NIH) NeuroBioBank (*n* = 11). Control pediatric cases were included that had an explained COD, resulting from accidents, homicide, or natural manner of death, and with FFPE or formalin fixed (in formalin less than 3 years) brain tissue available in the frontal cortex and hippocampus at the level of LGN. Control case exclusion criteria was as follows: significant neurological, pulmonary, and/or cardiac diagnoses. The adjudication for COD of SUDC and control cases was determined before proteomics analyses. The number of cases with each brain region available are detailed in Supplementary Table 1, online resource, as some regions were not evaluated due to block availability and tissue artifact. Case history for the 19 control and 19 SUDC cases is summarized in Table 1, detailed further in Tables 2, 3 and Supplementary Table 1, online resource. Group sizes were determined based on the number of cases with significant findings as previously reported [33, 50, 65], including our previous study in epilepsy cases with similar methods [60].

**Table 1:** Case history summary

Group	Cases	Mean Age at Death (yr)	Sex	Mean PMI (hr)	Mean Brain Weight (grams)
Control	19	2.7 ± 1.6	5 F/14 M	24 ± 10	1208 ± 142
SUDC	19	3.7 ± 3.7	10 F/9 M	37 ± 26	1267 ± 125
SUDC-noFS	8	5.0 ± 5.6	2 F/6 M	33 ± 22	1265 ± 168
SUDC-FS	11	2.8 ± 0.8	8 F/3 M	39 ± 29	1269 ± 90

Mean ± standard deviation is indicated

*FS* febrile seizures, *yr* years, *hr* hours, *PMI* post-mortem interval

**Table 2:** Control case history

Control case ID	Age (yr)	Sex	PMI (hr)	Brain weight (grams)	Cause of death
1	4	M	33	1222	Multiple stab wounds to chest
2	6	M	17	1495	Drowning
3	4	M	22	1350	Delayed complications of freshwater drowning
4	1	M	16	1010	Drowning
5	2	M	34	1310	Freshwater drowning
6	1	F	46	1091	Blunt force injuries
7	1	F	39	1016	Drowning
8	2	M	12	1100	Drowning
9	2	M	9	1243	Drowning
10	4	F	21	1502	Smoke inhalation
11	6	F	22	1323	Smoke inhalation
12	2	M	24	1080	Hanging
13	4	M	25	1290	Head and neck injuries
14	1.25	M	39	1188	Foreign object asphyxia
15	3	F	13	1170	Freshwater drowning
16	1.67	M	14	1132	Inhalation of combustion products, residential fire
17	1.83	M	24	1197	Asphyxia, neck compression
18	1.83	M	16	1143	Mechanical asphyxia
19	2	M	21	1090	Gunshot wound to chest

*yr* years, *hr* hours, *PMI* post-mortem interval

**Table 3:** SUDC case history

SUDC Case ID	Age (yr)	Sex	Other medical history	Febrile Seizure History	Apparent Terminal Activity	Found Body Position	PMI (hr)	Brain weight (grams)	Significant neuropathology
20	1.45	F	Fever 24 h	Simple	Sleep	Prone	55	1200	Mild hippocampal microscopic abnormalities; early acute hypoxic-ischemic neuronal damage
21	1.79	M	Fever 24 h, Mild motor/speech delay, autism spectrum	Simple	Sleep	Prone	35	1320	Mild hippocampal microscopic abnormalities and mild reactive changes
22	1.83	F	Fever 24–72 h, virus detected	Simple	Sleep	Prone	29	1180	
23	3.48	F		Simple	Sleep	Prone	30	1173	
24	1.48	F	Fever 24–72 h	None	Sleep	Prone	12	1241	
25	3.33	F	Speech delay	Complex	Sleep	Prone	9	1220	
26	2.90	F	Frequent URIs, pneumonia	Simple	Sleep	Prone	61	1170	
27	2.73	M		Simple	Sleep	Prone	112	1310	
28	1.85	M	Fever 24–72 h, virus detected	None	Sleep	Prone	20	1234	Left hippocampal asymmetry and dilated perivascular spaces
29	2.39	M		None	Sleep	Prone	18	1220	Bilateral hippocampal abnormalities, mild
30	2.58	M	Asthma, reactive airway disease, frequent URIs, Mild motor/speech delay, poor visual function	None	Sleep	Prone	15	1210	Hippocampal abnormalities, mild
31	2.88	F	Fever 24-72 h, Mild motor/speech delay	Simple	Sleep	Prone	38	1275	Mild reactive change
32	2.82	F	Fever 24-72 h, virus detected, mild sleep apnea, reactive airway disease	Complex	Rest	Prone	17	1286	
33	3.72	M	Reactive airway disease	Simple	Sleep	Supine	8	1430	
34	15.98	F	Asthma, reactive airway disease, respiratory infections	None	Sleep	Prone	38	1520	Focal cortical dysplasia, type IIA; hippocampal dysgenesis
35	1.18	M		None	Sleep	Side	26	945	
36	3.58	F	Fever 24-72 h, virus detected	Simple	Sleep	Prone	36	1400	Hippocampal abnormalities, mild
37	11.84	M	Asthma, multiple URIs	None	Sleep	Side	83	1390	Diffuse edema
38	2.61	M	Fever 72 h	None	Sleep	Prone	37	1358	Diffuse edema and hippocampal malrotation with variation in the thickness of the dentate gyrus

*URI* upper respiratory infection, *yr* years, *hr* hours, *PMI* post-mortem interval

### WES

Previous WES analyses in SUDC cases [22] were reviewed for APOE variants to determine significance in this population, as there is increased risk of seizure associated with APOE genotype [41, 44], impact on brain development [6], and poor outcome after traumatic brain injury [34]. Briefly, GATK Haplotype caller with depth ≥ 10 and alternate counts ≥ 5 was used to annotate variants. Average coverage was 77X. After annotation, each case was reviewed for APOE variants rs440446 (E3), rs429358 (E4), or rs7412 (E2). Heterozygosity was indicated at an allele frequency < 0.9.

### Laser capture microdissection (LCM)

After fixation in formalin, FFPE brain tissue blocks containing either the superior frontal gyrus of the frontal cortex or hippocampus (level of LGN) were sectioned at 8 µm and collected onto LCM compatible PET slides (Leica). Sections were stained with cresyl violet to localize regions of interest for LCM [15] and air dried overnight in a loosely closed container. LCM was used to individually microdissect 10 mm^2^ from the frontal cortex (gray matter layers I–IV) and hippocampal CA1-3, and 4 mm^2^ from the hippocampal DG into LC–MS grade water (Thermo Scientific). Microdissected samples were centrifuged for 2 min at 14,000* g* and stored at – 80 °C. LCM was performed at 5X magnification with a LMD6500 microscope equipped with a UV laser (Leica).

### Label-free quantitative mass spectrometry (MS) proteomics

Label-free quantitative MS was performed to identify differential protein expression, as described previously [43, 60]. Proteins were extracted and digested in each brain region separately in random batches of 16 samples according to S-Trap protocol. In brief, samples were supplemented with SDS (5% final), DTT (5 mM), TEAB (50 mM) and incubated two times for 30 min at 95 °C with 10 min sonication in water bath in between. Protein-SH groups were alkylated with iodoacetamide (15 mM, 1 h at RT in the dark). Samples were acidified with phosphoric acid (to final 1.2%) and proteins were precipitated by 6X (v/v) dilution in S-Trap buffer (90% MeOH in 100 mM TEAB) with subsequent loading onto micro-S-Trap spin columns where they were washed with S-Trap buffer and then digested with trypsin in 50 mM ammonium bicarbonate for 1 h at 47 °C (according to the manufacturer protocol). Resulting peptides were eluted from the columns, acidified with formic acid (FA) and subsequently desalted on in-house made C18 StageTips. Desalted peptide eluates were concentrated by vacuum evaporation on speedvac and resolubilized in 0.5% acetic acid (AcOH) prior to LC–MS/MS analyses. LC separation was performed online on EASY-nLC 1000 (Thermo Scientific) utilizing Acclaim PepMap 100 (75 µm × 2 cm) precolumn and PepMap RSLC C18 (2 µm, 100A × 50 cm) analytical column. Peptides were gradient eluted from the column directly into a Q Exactive HF-X mass spectrometer using a 160 min acetonitrile (ACN) gradient from 5 to 26% B in 115 min followed by a ramp to 40% B in 20 min and final equilibration in 100% B for 15 min (A = 2% ACN 0.5% AcOH/B = 80% ACN 0.5% AcOH). Flowrate was set at 200 nl/min. High-resolution full MS spectra were acquired with a resolution of 120,000, an AGC target of 3e6, with a maximum ion injection time of 32 ms, and a scan range of 400–1600 m/z. Following each full MS scan, 20 data-dependent HCD MS/MS scans were acquired at a resolution of 7500, AGC target of 2e5, maximum ion time of 32 ms, one microscan, 1.4 m/z isolation window, fixed first mass of 200 *m/z*, normalized collision energy (NCE) of 27 and dynamic exclusion of 45 s.

### Proteomics computational analysis

MS data were analyzed as previously described [43, 60]. In brief, MS data were analyzed using MaxQuant software version 1.6.3.42 and searched against the SwissProt subset of the *H. sapiens* Uniprot database (http://www.uniprot.org/) containing 20,365 entries (February 2019 release) to which 248 common laboratory contaminants were added. Database search was performed using the Andromeda search engine integrated into the MaxQuant environment. The enzyme specificity was set to trypsin with the maximum number of missed cleavages set to 2. The precursor mass tolerance was set to 20 ppm for the first search used for non-linear mass re-calibration and then to 6 ppm for the main search. Oxidation of methionine was searched as variable modification; carbamidomethylation of cysteines was searched as a fixed modification. The false discovery rate (FDR) for peptide, protein, and site identification was set to 1%, the minimum peptide length was set to 6. To transfer identifications across different runs, the ‘match between runs’ option in MaxQuant was enabled with a retention time window of 1 min. Protein quantification was performed with built-in MaxQuant LFQ algorithm with the following settings: minimum ratio count of 2, “fastLFQ” option enabled, minimum/average number of neighbors 3/6. Subsequent data analysis was performed in either Perseus (http://www.perseus-framework.org/) or using R environment (http://www.r-project.org/). MS RAW files were uploaded to the MassIVE repository (https://massive.ucsd.edu/) with the following dataset ID MSV000088611.

### Proteomics statistical analyses

The protein expression matrix (*n* = 3,798) was filtered to contain only proteins that were quantified in at least 50% of cases in ≥ 1 group (SUDC or control) in each brain region (*n* = 3,012 frontal cortex, *n* = 2,954 DG, *n* = 2,958 hippocampal CA1-3). In the frontal cortex, 1487/3012 proteins were detected in all cases, 2936/3012 were detected in half of the cases regardless of group, and 3012/3012 were detected in at least half of the cases in one group. In the dentate gyrus, 1395/2954 proteins were detected in all cases, 2873/2954 were detected in half of the cases regardless of group, and 2954/2954 were detected in at least half of one group. In the hippocampal CA1-3, 1611/2958 proteins were detected in all cases, 2845/2958 were detected in half of the cases regardless of group, and 2958/2958 were detected in at least half of one group. For Principal Component Analysis (PCA), missing values were imputed from the normal distribution with a width of 0.3 and downshift of 1.8 (relative to measured protein intensity distribution). A Student’s two-sample *t* test was performed in Perseus v. 1.6.2.3 (http://www.perseusframework.org/) [64] on imputed data to detect significant changes in protein expression between SUDC and control cases. Thresholds set for p value were adjusted to provide a false discovery rate (FDR) below 5% (permutation-based FDR with 250 data randomizations). A comparison of the proteins detected common to each region, as well as the significant proteins, were evaluated by Venn diagram generated from InteractiVenn [25]. Cell type-specific annotations were included in Supplementary Table 1–3, online resource, and on volcano plots in Fig. 2a–c, from a reference data set [40] and as described previously [43, 60]. Annotations were included when a protein had only one associated cell type and when the annotation included more than one associated cell type but were only neuronal proteins, for a total of 1066 possible annotations.

### Pathway analysis

Ingenuity Pathway Analysis (IPA, Qiagen) was used to determine the signaling pathways associated with differentially expressed proteins. All detected proteins were included in the data set for each brain region, including the UniProtID, fold change, and *p* value. A core analysis was performed in each brain region for proteins at a FDR < 5%. Pathways were considered enriched at a *p* value of overla*p* < 0.05 and to be activated/inhibited as a result of combined protein fold changes in a pathway as reflected by a |*z* score|≥ 2. Coronavirus pathogenesis pathway is included in the supplementary table output, but not included in the total number of significantly associated pathways as the altered proteins may be non-specific to this pathway (four increased ribosomal proteins in DG) and this study was performed before the COVID-19 pandemic.

### Weighted gene correlation network analysis (WGCNA)

WGCNA was performed to determine protein correlations with SUDC clinical variables (detailed in Supplementary Table 1, online resource) in the R environment with the *WGCNA* package for blockwiseModules with defaults except where noted, similar to previously described [32, 62]. Soft threshold power beta was determined at *R*^2^ = 0.8. The power used for each brain region: frontal cortex = 6, DG = 9, and hippocampal CA1-3 = 5. Gene ontology (GO) annotations for modules was determined following WGCNA with the *anRichment* package in the *R* environment with Entrez IDs against the human GOcollection. GO annotations were considered with an FDR < 5% and associated with at least five proteins.

## Results

### Case history

The SUDC group included cases with febrile seizure history (SUDC-FS, 57.9%) and without (SUDC-noFS, 42.1%; Tables 1, 3). Frequency of neuropathology hippocampal (NP HP, *n* = 8/19) findings were not different in SUDC-FS cases compared to SUDC-noFS (*p* = 0.18, Fisher’s exact test), nor was terminal fever at 72 h before death (*n* = 9/19, *p* = 0.65), terminal fever at 24 h before death (*n* = 6/19, *p* > 0.99), or virus detection (*n* = 4/19, *p* = 0.60). Not all cases with fever 24 h prior to death had virus detected at autopsy, but all four cases with virus did have fever 24 h prior to death. In those cases with virus detected, there was no additional clinical history or symptoms to suggest that viral infection was related to COD. Several SUDC cases had a clinical history of motor/speech delay (*n* = 4) or pulmonary disease (*n* = 6). The control cases all had an explained COD (Table 2), with no differences from the SUDC group in age (*p* = 0.27), sex (*p* = 0.18), brain weight (*p* = 0.18), or post-mortem interval (PMI; *p* = 0.069).

Due to the increased risk of seizure associated with APOE genotype [41, 44] and its impact on brain development [6], we evaluated APOE genotype in SUDC cases from previous WES [22]. Among the 19 SUDC cases, there was 1 APO E2/E3 case, 11 E3/E3, 5 E3/E4, and 2 E4/E4. E4 carriers (*n* = 7/19, 37%) were not more common among SUDC-FS (*p* > 0.99) nor in cases with motor/speech delay (*p* = 0.12).

### Protein differential expression

**Fig. 1:**
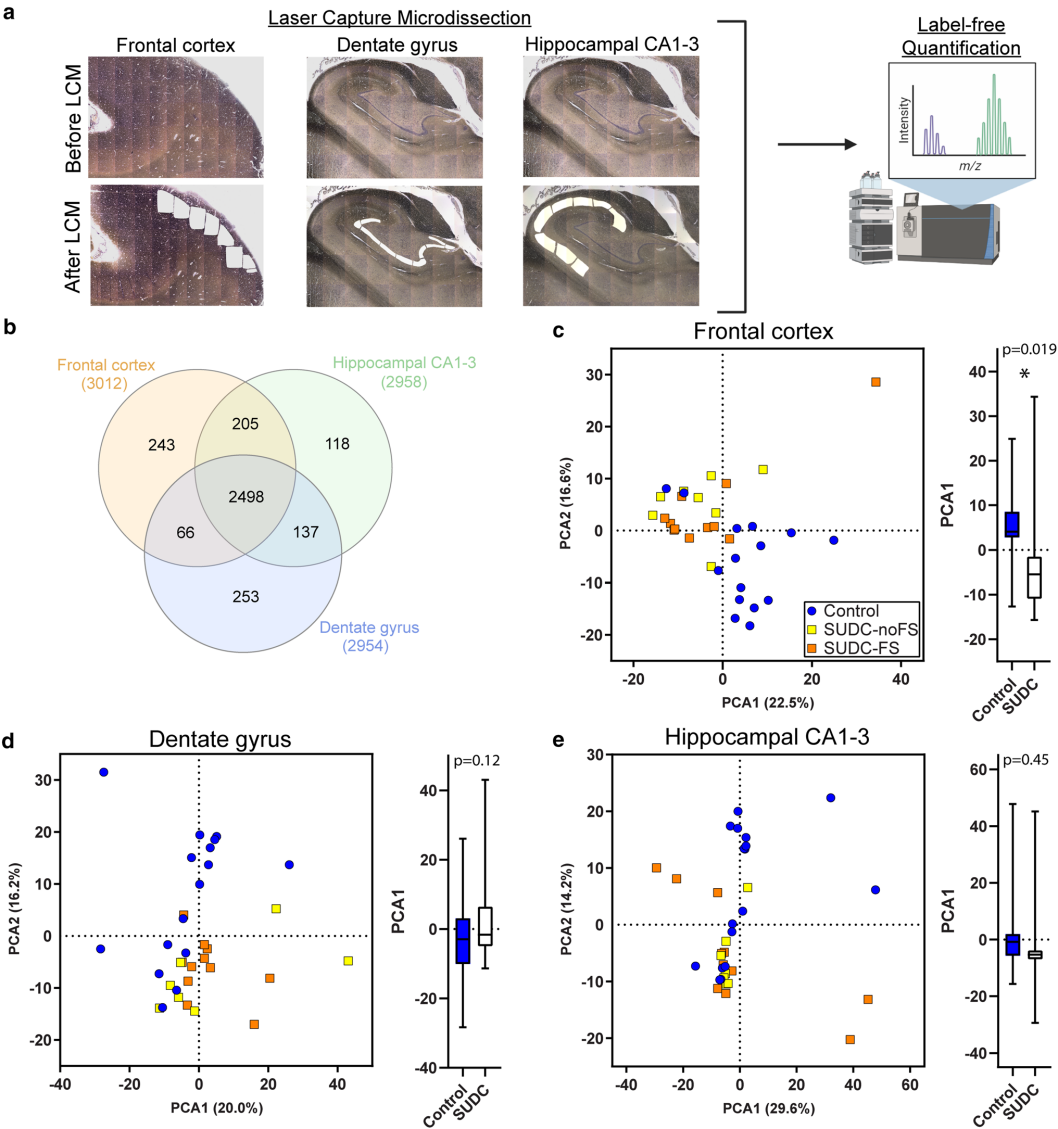
Detected proteins and PCA from proteomics analyses in frontal cortex, dentate gyrus, and hippocampal CA1-3.** a** Number of proteins detected in each brain region by label-free quantitative MS is indicated, with an overlap of 2498 proteins detected in all brain regions. **b** The PCA in the frontal cortex shows separation of control (*n* = 15) and SUDC (*n* = 19) cases. In the hippocampus, the variation is indicated for **c** the dentate gyrus (control *n* = 16, SUDC *n* = 18) and **d** the hippocampal CA1-3 (control *n* = 17, SUDC *n* = 18). An analysis of group separation in PCA1 for each brain region is depicted by a box plot with bars indicating minimum and maximum values, evaluated by an unpaired two-tailed t test. *PCA1* principal component analysis 1, *PCA2* principal component analysis 2

Protein differential expression analysis was evaluated in SUDC (*n* = 19) and control cases with explained COD (*n* = 19) from autopsy brain tissue with label-free quantitative MS in the microdissected frontal cortex, DG, and hippocampal CA1-3 (Fig. 1a). Label-free quantitative MS identified 3798 proteins in the cases analyzed, detected in at least 50% of cases the SUDC or control groups in frontal cortex (*n* = 3012), DG (2954), and hippocampal CA1-3 (2958; Supplementary Table 3–5, online resource, Fig. 1b). Among all 3 regions, 2498 proteins were commonly detected in all brain regions. PCA showed a significant separation of SUDC and control cases in frontal cortex (*p* = 0.019, Fig. 1c). There was no segregation of SUDC from control in the DG (*p* = 0.12, Fig. 1d) or hippocampal CA1-3 (*p* = 0.45, Fig. 1e), nor by febrile seizure history in any region. Furthermore, age and PMI did not contribute to PCA1 in any brain region by multiple variable linear regression analysis (Supplementary Table 2, online resource).

**Fig. 2:**
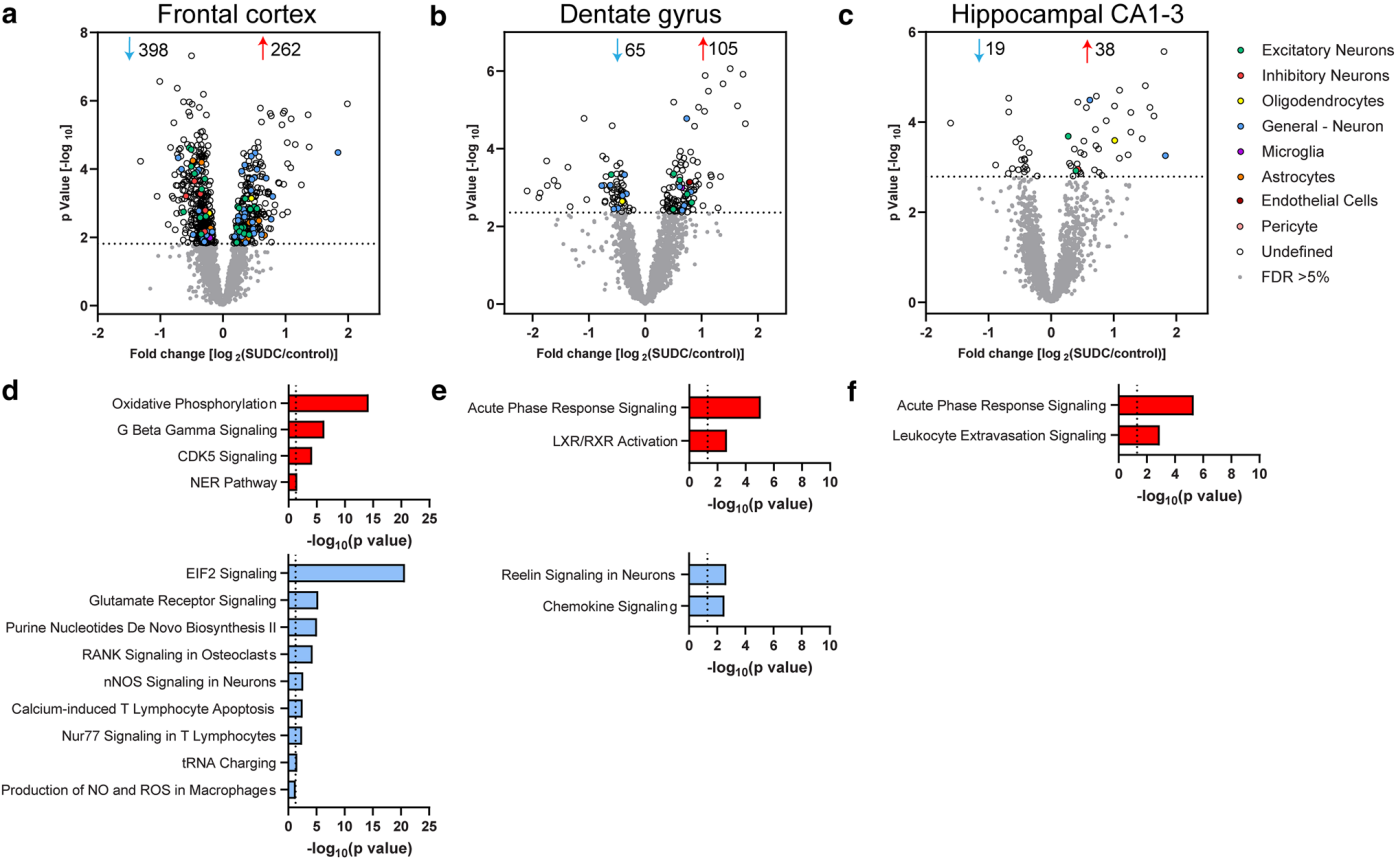
Differential proteomic expression analyses in frontal cortex, dentate gyrus, and hippocampal CA1-3. **a** Volcano plots indicate significantly different proteins in SUDC when compared to control cases after a student’s two-tailed *t* test with permutation correction at a 5% FDR in **a** frontal cortex, **b** dentate gyrus, and **c** hippocampal CA1-3. Cell type-specific protein annotation is indicated, with the “General—Neuron” annotation including both excitatory and inhibitory neuron annotations. IPA pathway enrichment analysis of the significantly altered proteins identified the indicated pathways that are activated (red) or inhibited (blue) with a significant z score ( ≥|2|) and p value of overlap (*p* < -log_10_(0.05)) for **d** frontal cortex, **e** dentate gyrus, and **f** hippocampal CA1-3

With a student’s t test followed by permutation-based FDR at 5%, there were significant differences between SUDC and control cases in 660 proteins of frontal cortex, 170 in DG, and 57 in hippocampal CA1-3 (Fig. 2a–c, Supplementary Table 3, 4, 5, online resource). The top 20 most significant proteins in each brain region are summarized in Supplementary Tables 6–8, online resource. Among all brain regions, there were six proteins commonly altered (Supplementary Table 9, online resource). After cell type annotation of proteins, the majority of proteins are “undefined” in all brain regions and likely expressed by multiple cell types or association is unknown. After “undefined” the most abundant annotation for significant proteins in each region was for neuronal proteins, with 10.8% in frontal cortex, 10.6% in DG, and 8.8% in the hippocampal CA1-3 region. Neuronal proteins (“neuron,” “excitatory,” and “inhibitory”) were enriched in the DG (*p* = 0.0005, Fisher’s exact test), while no other cell type annotations were enriched in the other brain regions.

### Pathway analysis

Pathway analysis of the significantly altered proteins in frontal cortex indicated that there were 238 signaling pathways associated with the 660 proteins (*p* value of overlap *p* < 0.05) and that 13 of these pathways were significantly impacted by fold change as reflected by the z score (|*z*|≥ 2; Supplementary Table 10, online resource). Top pathways included activation of oxidative phosphorylation (*p* = 6.3 × 10^–15^, *z* = 4.08) and inhibition of EIF2 signaling (*p* = 2.0 × 10^–21^, *z* = − 2.56; Fig. 2d). Interestingly, glutamate receptor signaling was decreased (including increased GLS, GLUL, GRM5, SLC17A7, SLC1A2 and decreased GRIA1, GRIA3, GRID1). Among the 13 signaling pathways, 5 pathways (oxidative phosphorylation, EIF2 signaling, NER pathway, purine nucleotides de novo biosynthesis II, tRNA charging) included unique proteins that did not overlap with other signaling pathways. The remaining eight pathways shared some of the same significant proteins.

In the DG, there were 54 signaling pathways associated with the 170 significant proteins and 4 of these pathways were significantly impacted by fold change (|*z*|≥ 2; Supplementary Table 11, online resource). Top pathways included activation of acute phase response signaling (*p* = 8.5 × 10^–6^, *z* = 2.65) and inhibition of reelin signaling in neurons (*p* = 2.2 × 10^–3^, *z* = − 2.24; Fig. 2e). There were shared significant proteins among all four pathways. Reelin signaling was associated with increased CFL1 and decreased GRIN1, PDK2, RHOA, and SRC.

In the hippocampal CA1-3, there were 67 signaling pathways associated with the 57 significant proteins and 2 of these pathways were significantly impacted by fold change (|*z*|≥ 2; Supplementary Table 12, online resource). The top pathway was activation of acute phase response signaling (*p* = 4.7 × 10^–6^, *z* = 2.00; Fig. 2f). Acute phase response signaling was associated with increased A2M, APOA1, FGA, FGB, FGG in both hippocampal regions, as well as increased C3, C4A/C4B, SERPINA1, and SOD2 in DG and increased TF in CA1-3. There was no overlap of proteins in the two altered pathways in the hippocampal CA1-3.

### Brain region comparative analysis

**Fig. 3:**
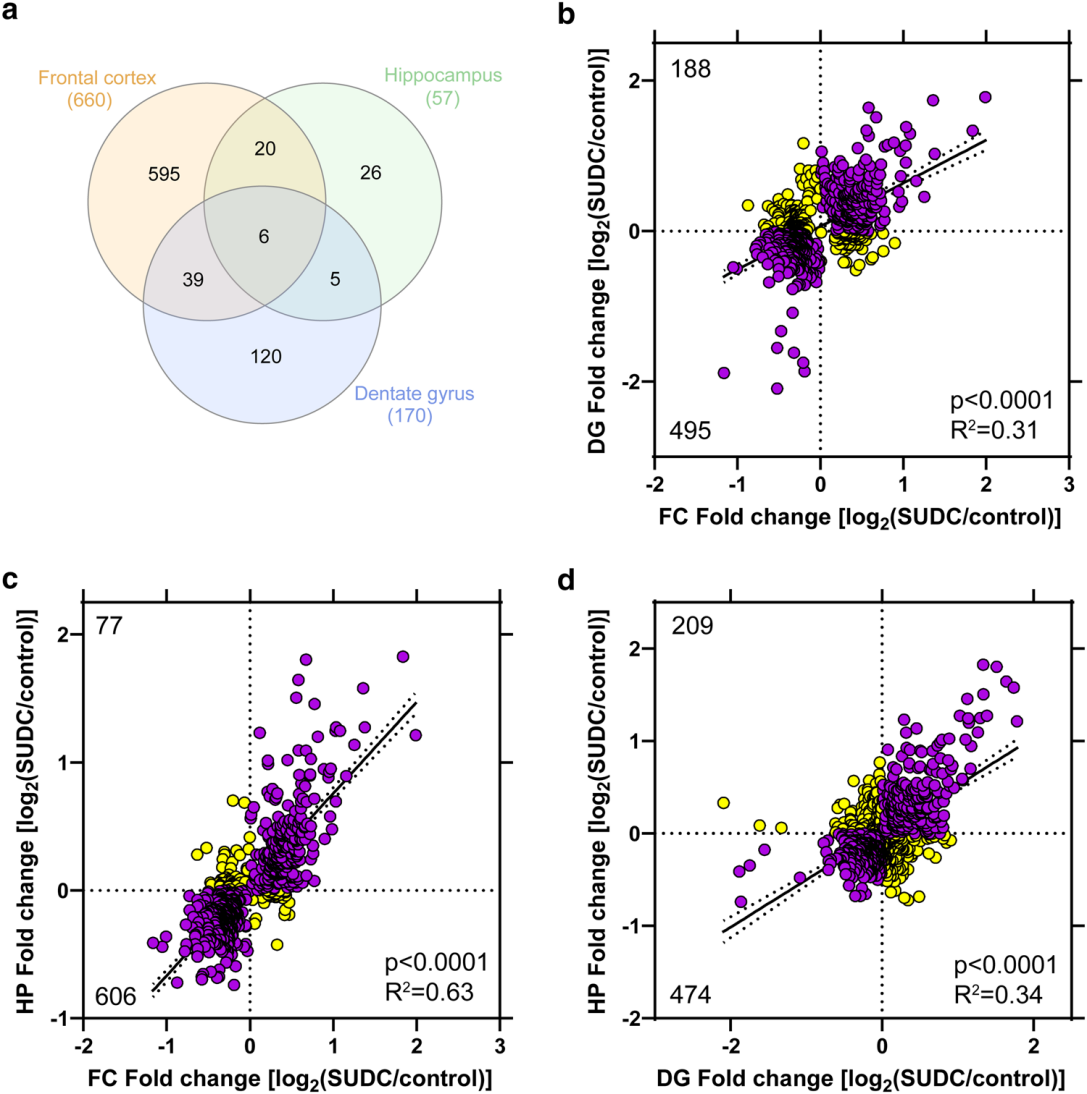
Inter-regional correlation analyses. **a** Among significant proteins in each brain region analyzed, there were 6 proteins significantly altered in all 3 brain regions. There were more similarities when comparing 2 brain regions, with the most similar proteins between the frontal cortex and dentate gyrus (39 proteins) and highest percentage of shared proteins between the frontal cortex and hippocampal CA1-3 (46% hippocampal CA1-3 proteins). **b** Of the significant proteins detected in all regions (*n* = 683 significant and detected/811 total significant), a correlation analysis of frontal cortex and dentate gyrus indicated a significant positive correlation (*p* < 0.0001, *R*^2^ = 0.31) with 495 proteins (72.5% proteins) changing in the same direction (up/down, purple) and 188 proteins changing in the opposite direction (i.e. up in frontal cortex and down in dentate gyrus; yellow). **c** Of the significant proteins detected in all regions (*n* = 683), a correlation analysis of frontal cortex and hippocampal CA1-3 indicated a significant positive correlation (*p* < 0.0001, *R*^2^ = 0.63) with 606 proteins (88.7%) changing in the same direction (up/down, purple) and 77 proteins changing in the opposite direction (yellow). **d** Of the significant proteins detected in all regions (*n* = 683), a correlation analysis of dentate gyrus and hippocampal CA1-3 indicated a significant positive correlation (*p* < 0.0001, *R*^2^ = 0.34) with 474 proteins (69.4%) changing in the same direction (up/down, purple) and 209 proteins changing in the opposite direction (yellow)

Among all brain regions, there were six proteins commonly altered (Fig. 3a, Supplementary Table 9, online resource). There were no signaling pathways associated with these six proteins (p value of overlap *p* < 0.05). To determine whether additional proteins were trending in the same direction globally across brain regions, correlation analyses were performed and indicated that of all significant proteins detected in all regions (*n* = 683 detected out of 811 total significant proteins), there is a significant correlation with all other brain regions analyzed with the best correlation between frontal cortex and hippocampal CA1-3 (Fig. 3b–d). Specifically, there is an overlap in proteins correlating in the same direction (up/down) when comparing frontal cortex and the DG (72.5% proteins, Fig. 3b), frontal cortex and the hippocampal CA1-3 (88.7% proteins, Fig. 3c), and the DG and hippocampal CA1-3 (69.4% proteins, Fig. 3d).

### Correlation to clinical history

**Fig. 4:**
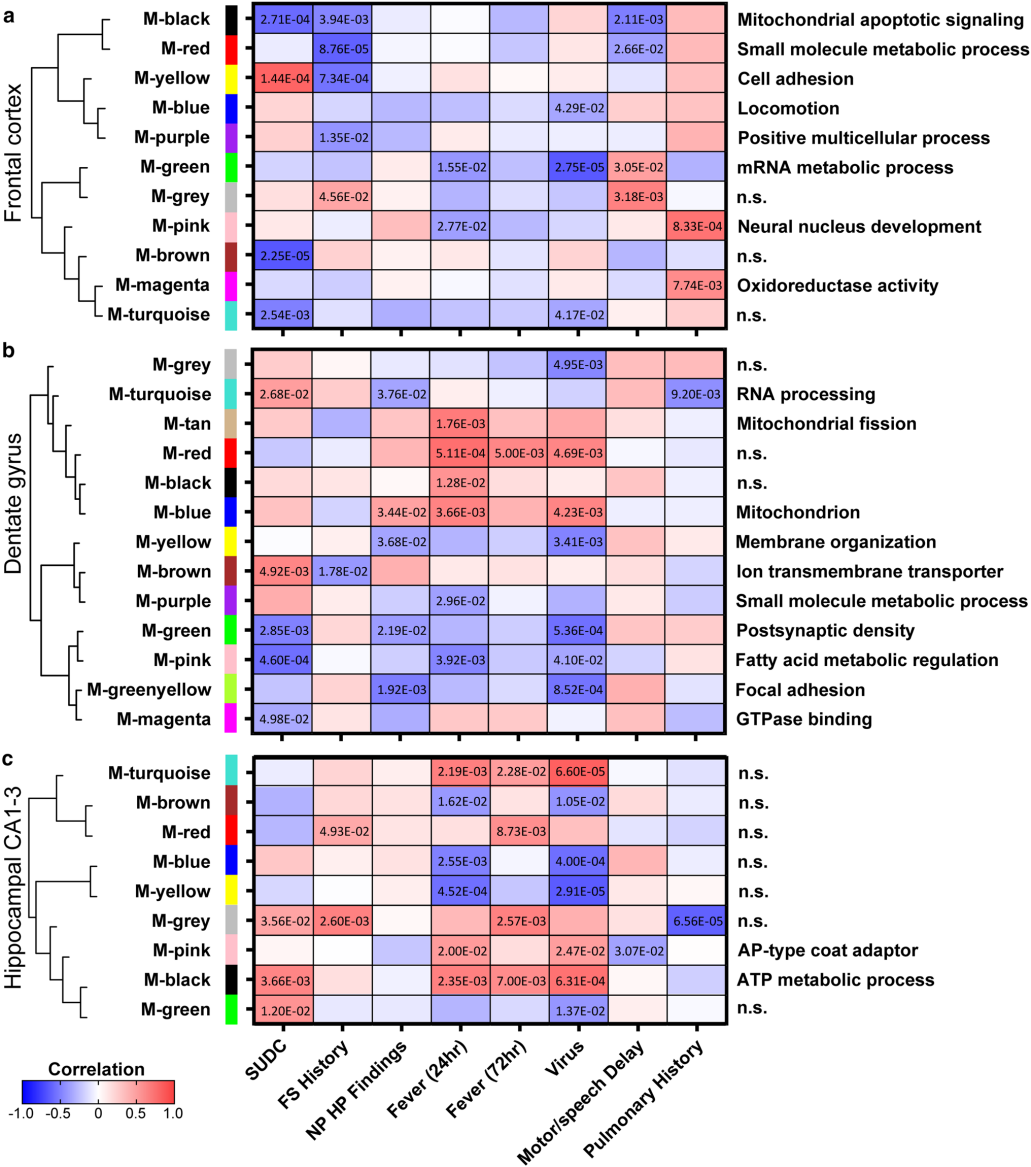
WGCNA of clinical variables in frontal cortex, dentate gyrus, and hippocampal CA1-3. A correlation analysis of clinical variables to proteomics indicated significant modules and associated GO annotations in the **a** frontal cortex **b** dentate gyrus, and **c** hippocampal CA1-3. Modules are clustered by eigenprotein adjacency (relatedness to other modules) on the left. Name of module is indicated by “M-color” and corresponding color block. *P* values are indicated for those modules with *p* < 0.05 correlation. Positive correlation is indicated in red and negative correlation in blue. Top module GO annotations are noted on the right (FDR < 5% with at least 5 proteins) and detailed in Supplementary Table 13–15, online resource. Several modules did not have a significant GO annotation and are noted as “n.s.” *FS* febrile seizure, *NP HP* findings neuropathology hippocampal findings

To determine whether clinical history (Table 3) correlated to protein changes, a WGCNA was performed in each brain region (Fig. 4, Supplementary Fig. 1, online resource). Among all brain regions, the most significant modules after the SUDC group (first column) correlated to detected virus at autopsy (*n* = 4/17). In the frontal cortex, all clinical variables correlated to at least one module except NP HP findings and fever within 72 h (Fig. 4a). In the DG, all clinical variables correlated to at least one module except motor/speech delay (Fig. 4b). Interestingly, the cases with virus detected had similar significant modules identified in the DG of cases with NP HP findings. Some of these NP HP finding-related pathways were also associated with other variables in which cases had pulmonary history and/or fever within 24 h. In the hippocampal CA1-3, all clinical variables correlated to at least one module except NP HP findings (Fig. 4c). Furthermore, there were several clinical variables that had an opposite correlation to that which was identified in the SUDC group overall, i.e. M-yellow in frontal cortex of SUDC and FS history. The top GO annotations (FDR < 5% with at least five proteins) associated with each module are noted (Fig. 4) and detailed further in Supplementary Table 13–15, online resource, with some modules having no significant (“n.s.”) GO annotation.

### Comparison of SUDC to epilepsy

To identify proteomic similarities between SUDC and epilepsy brain tissue, we performed a comparative analysis to our previous dataset [60] from the same brain regions. Most common protein changes in SUDC and epilepsy were identified in the frontal cortex, with 82/660 proteins overlapping (Supplementary Fig. 2a, online resource). There were fewer commonalities in the DG and hippocampal CA1-3 (Supplementary Fig. 2b–c, online resource). Among the 82 common proteins in frontal cortex, 15/82 proteins had a fold change relative to controls in the same direction and 67/82 had a fold change in the opposite direction (i.e. increased in SUDC and decreased in epilepsy). There was a negative correlation for these 82 proteins between SUDC and epilepsy (*p* < 0.0001, *R*^2^ = 0.39; Supplementary Fig. 2d, online resource). The mitochondrial enzyme COX6B1 had the largest fold change in both SUDC and epilepsy, which was decreased in both groups.

## Discussion

We evaluated differential expression of proteins in microdissected frontal cortex, hippocampal DG, and CA1-3 in SUDC compared to control cases with an explained COD. We identified protein changes in all brain regions analyzed, particularly in the frontal cortex. Top signaling pathways associated with the altered proteins in frontal cortex indicated increased activation of oxidative phosphorylation and G beta gamma signaling and inhibition of EIF2 signaling and glutamate receptor signaling. In the hippocampal regions, top pathways included activation of acute phase response and inhibition of reelin signaling. A comparison of proteins across different brain regions indicated some similarities in global protein changes and many brain region specific changes. Furthermore, WGCNA identified protein clusters that correlated to clinical history, most significantly to cases with detected virus at autopsy.

There were multiple signaling pathways altered in the frontal cortex, a region that has not typically been implicated in SUDC nor described to have detectable neuropathology [26, 37, 49]. Although hippocampal abnormalities have been implicated in SUDC [26, 37], some studies question the significance of these findings and the normal range in variation of morphological differences in this region [1, 42, 48, 61]. The most significantly altered pathway in frontal cortex was inhibition of EIF2 signaling, which is involved in protein translation, along with the related pathway tRNA charging that was also inhibited. Reduced translation may be associated with a cellular stress response to external stimuli, as occurs after hypoxia or DNA damage, to conserve cellular energy [45]. There was activation of the NER (nucleotide excision repair) pathway, associated with DNA damage repair. DNA damage can result in increased activation of oxidative phosphorylation [2], as we observed. However, there was inhibition of purine nucleotides de novo biosynthesis II, which is necessary for DNA synthesis and cellular growth, although may require increased translation [66]. An alteration of the observed pathways and potential etiologies that result in DNA damage should be investigated further, particularly evaluating cell type-specific vulnerability (i.e. glutamatergic neurons), subregional impact (i.e. laminar layer I vs. IV), protein candidate or pathway functionality in the context of SUDC risk (i.e. cell or animal model), and additional brain regions with reciprocal connections to the frontal cortex (i.e. proteomics in dysfunctional autonomic brainstem nuclei).

Of the 13 altered pathways in frontal cortex, 8 pathways had shared proteins indicating an overlap of signaling pathways (i.e. general kinases and phosphatases). Glutamate receptor signaling was inhibited, with decreases in glutamate receptors and enzymes involved in glutamate metabolism, which may indicate a compensatory response to minimize hyperactivity and energy demands. Glutamate is a potent activator of nNOS signaling in neurons (important in neuronal plasticity), which was inhibited. CDK5 signaling was activated with increased CDK5 protein, which can facilitate neuronal migration and neurite outgrowth. The phosphorylation of proteins in this signaling pathway should be investigated further to understand the implications of this pathway in the context of the other altered pathways. Furthermore, there were shared proteins among the CDK5 signaling pathway and G beta gamma signaling, production of NO and ROS in macrophages, RANK signaling in osteoclasts, and Nur77 signaling in T lymphocytes. These pathways should be investigated further, i.e. including the unique proteins altered in these pathways to verify functional implications. Several inflammatory response pathways were inhibited (e.g., calcium-induced T lymphocyte apoptosis, Nur77 signaling in T lymphocytes, production of NO and ROS in macrophages) that deserve further investigation.

Hippocampal regions showed activation of an inflammatory response (acute phase response, LXR/RXR activation, leukocyte extravasation) and reduced chemokine signaling. Reelin signaling was inhibited in DG. Some proteins in this pathway overlap with the chemokine signaling pathway. Defects in reelin signaling are suggested in SUDC cases with hippocampal abnormalities [26, 37]. Reelin is also related to abnormal granule cell migration and dispersion in epilepsy patients [21], after seizure induction in animal models [5, 54, 63], and a reelin knockout mouse model had developmental defects [19, 53]. Further investigation is required to identify the etiologies underlying these pathway changes, including how the neuronal projections between the hippocampus and frontal cortex may influence altered signaling pathways, i.e. whether a hippocampal inflammatory response results in decreased energy demands in cortex by limiting glutamate receptor signaling, etc.

There were few global protein changes identified. In all brain regions analyzed, HBA1, HBB, and FGB were increased. TJP1 (ZO-1) was significantly decreased in frontal cortex only, indicating changes in vascularization, BBB permeability, and/or inflammation. Furthermore, CADM3 and ACTC1 were increased in all regions, indicating changes in actin stability/motility and neural migration/development. In addition, HMGCS1 was decreased, which can reduce cholesterol synthesis associated with cellular energy production. There were further proteins trending in a correlation analysis among regions as well, indicating that many of the significant signaling pathways identified may be impacted in other brain regions, but to a lesser extent.

WGCNA identified several protein clusters correlated to SUDC clinical history. The strongest correlation in all brain regions was with cases that had detected virus at autopsy. The modules associated with virus detected also overlapped with modules identified in the DG of cases with NP HP findings. FS history was associated with several significant modules in frontal cortex, 1 module in the DG (decreased ion transmembrane transporter), and 2 modules in CA1-3 with no significant GO annotation. To better characterize cases with the clinical history observed in the SUDC cohort, additional cases should be investigated further for those clinical variables with few cases, including those cases with positive post-mortem virology that may have occurred ante-mortem or post-mortem.

We evaluated APOE genotypes in SUDC cases. There was a higher prevalence for carriers of the E4 allele (*n* = 7/19, 37%) in the SUDC cohort than in other prior studies [6, 7, 9]. Most studies have a more biased evaluation of E4 allele frequency in adults, particularly with a history of dementia [7, 9]. In pediatric cases, E4 allele carrier frequency was observed in 26% (*n* = 310/1187) [6]. In pediatric cases older than 3 years of age (*n* = 1187), brain development (including hippocampal volume) and cognitive abnormalities were associated with the E4/E4 and E2/E4 genotypes [6]. Traumatic brain injury in pediatric cases with the E4 allele were also associated with poor outcome when compared to cases without E4 [34]. Furthermore, seizures are more common in individuals with the E4 allele [41, 44]. However, we did not observe increased prevalence of E4 carriers among cases with FS or motor/developmental delay. Increased APOA1 was associated with activated acute phase response in the hippocampus and altered fatty acid metabolism in the DG. APOE is mainly expressed by astrocytes and helps repair and remodel membranes, although the E4 allele also alters brain metabolism and may impair repair mechanisms [46]. The anti-inflammatory mechanism mediated by APOA1 depends on APOE expression [18]. The influence of the E4 allele on early development may warrant further investigation, particularly in the context of inflammation, febrile seizures, and in combination with other genetic variants or external factors.

Although ours is the first proteomics study in SUDC, protein differences in brain tissue were examined in SUID cases. No proteomics analyses have been performed in the hippocampus or cortex of SUID cases, but in the medulla-binding studies and proteomics identified decreased binding of serotonin and altered protein expression in the serotonin signaling pathway [3, 16, 36]. Specifically, there was decreased serotonin 5HT1A receptor binding in the medulla of some SUID cases [16, 36]. In cases with reduced 5HT1A receptor binding, serotonin, and TPH2 in several medullary nuclei, there were also decreased 14-3-3 isoforms (YWHAG, H, B, Q), also known as Tyrosine 3-Monooxygenase/Tryptophan 5-Monooxygenase Activation Protein [3]. 14-3-3 proteins are involved in multiple cellular functions, including binding phosphorylated TPH2 and stabilizing this rate limiting enzyme in serotonin synthesis [31]. In the current study, YWHAE was decreased 1.3-fold (*p* = 1.54 × 10^–3^) only in the frontal cortex, several related proteins were detected but not altered in all regions (YWHAB, H, G, Q, Z, MAOA), and some serotonergic proteins were not detected in any region likely due to differential brain region expression and detection limitation by technique (TPH2, 5HT1A, 5HT2A, SERT). In addition to altered serotonergic signaling, increased inflammatory processes have been identified in SUID (sudden infant death syndrome, SIDS). A previous study found increased APOA1, A2M, and complement C3 in serum from SIDS cases [17], similar to our hippocampal findings in SUDC. These inflammatory and serotonergic proteins deserve further investigation in SUDC, particularly in the brainstem.

In addition to parallels in mechanism of death in SUDC and SUDEP [27, 28, 37, 49], altered serotonergic signaling in brainstem and other regions is postulated to contribute to SUDEP risk [43, 51, 55–58]. We evaluated the same brain regions in SUDEP, but did not identify differences [43]. In non-SUDEP epilepsy, we identified differences in adult cases [60], although with few similarities to SUDC (Supplementary Fig. 2, online resource). Differences between SUDC and epilepsy were apparent in the enriched signaling pathways, with inhibited EIF2 signaling and activated oxidative phosphorylation in the frontal cortex of SUDC while the opposite occurred in epilepsy. In the hippocampal CA1-3 region, leukocyte extravasation was activated in SUDC and the opposite occurred in epilepsy. There was no overlap of pathways in the DG. Future studies should compare to additional epilepsy cases, including specific epilepsy syndromes and pediatric cases, as well as non-epilepsy sudden death.

There were several limitations in our study. There is lower detection of large membrane proteins with the technique used. WES was performed in SUDC cases, and not in control cases. Potential variants of unknown significance (VUS) would require further investigation in all cases. Exome sequencing in brain tissue was not evaluated to determine whether there were de novo somatic variants in any cases. Among the SUDC cases, there may be heterogeneous etiologies associated with mechanism of death. The SUDC cases were acquired from referrals to the SUDCRRC, and control cases were obtained from those same collaborations as well as those cases referred to the NIH NeuroBioBank. Furthermore, cases were acquired from multiple sources with varying fixation and processing procedures, however, we were able to robustly identify proteomic alterations despite potential variability related to these differences.

Overall, we identified protein changes in SUDC as well as specific changes related to clinical variables in frontal cortex and hippocampus, indicating a need for follow-up analyses in a larger group of well-characterized cases for clinical history of interest, mechanistic studies, and analyses of additional brain regions with the aim of understanding and reducing SUDC risk.

## Supplementary Information

Below is the link to the electronic supplementary material.

Supplementary file1 (DOCX 221 kb)

Supplementary file2 (XLSX 5022 kb)
