# Supplementary material for: Proteomic differences in hippocampus and cortex of sudden unexplained death in childhood
Source: Acta Neuropathol. 2022 Mar 25;143(5):585–99. doi: 10.1007/s00401-022-02414-7 (PMC8953962; doi:10.1007/s00401-022-02414-7)

**Supplementary Materials:**

**Proteomic Differences in Hippocampus and Cortex of Sudden Unexplained Death in Childhood**

**Authors:** Dominique F. Leitner ^a,b^, Christopher William ^b,c^, Arline Faustin^b,c,d^, Manor Askenazi^e^, Evgeny Kanshin^f^, Matija Snuderl^c^, Declan McGuone^g^, Thomas Wisniewski^b,c,d,i^, Beatrix Ueberheide^b,f,h^, Laura Gould^b,j^, Orrin Devinsky^a,b^

^a^ Comprehensive Epilepsy Center, NYU Grossman School of Medicine, New York, NY, USA

^b^ Department of Neurology, NYU Grossman School of Medicine, New York, NY, USA

^c^ Department of Pathology, NYU Grossman School of Medicine, New York, NY, USA

^d^ Center for Cognitive Neurology, NYU Grossman School of Medicine, New York, NY, USA

^e^ Biomedical Hosting LLC, Arlington, MA, USA

^f^ Proteomics Laboratory, Division of Advanced Research Technologies, NYU Grossman School of Medicine, New York, NY, USA

^g^ Department of Pathology, Yale School of Medicine, CT, USA

^h^ Department of Biochemistry and Molecular Pharmacology, NYU Grossman School of Medicine, New York, NY, USA

^i^ Department of Psychiatry, NYU Grossman School of Medicine, New York, NY, USA

^j^ Sudden Unexplained Death in Childhood Foundation, NJ, USA

**Corresponding author:**

Orrin Devinsky

Comprehensive Epilepsy Center

Department of Neurology

NYU Langone Health and School of Medicine

New York, NY, USA

Email: [Orrin.Devinsky@nyulangone.org](mailto:Orrin.Devinsky@nyulangone.org)

**Tables:**

**Supplementary** **Table 1. Extended Case History**

**Supplementary** **Table 2. Case History Regression**

**Supplementary** **Table 3. LC-MS/MS Frontal Cortex**

**Supplementary** **Table 4. LC-MS/MS Dentate Gyrus**

**Supplementary** **Table 5. LC-MS/MS Hippocampal CA1-3**

**Supplementary** **Table 6. Top 20 Proteins Differentially Expressed in the Frontal Cortex**

**Supplementary** **Table 7. Top 20 Proteins Differentially Expressed in the Dentate Gyrus**

**Supplementary** **Table 8. Top 20 Proteins Differentially Expressed in the Hippocampus**

**Supplementary** **Table 9. Significant Proteins Common to All Brain Regions**

**Supplementary** **Table 10. IPA Pathways in the Frontal Cortex**

**Supplementary** **Table 11. IPA Pathways in the Dentate Gyrus**

**Supplementary** **Table 12. IPA Pathways in the Hippocampal CA1-3**

**Supplementary** **Table 13. WGCNA GOanRichment Analysis in the Frontal Cortex**

**Supplementary** **Table 14. WGCNA GOanRichment Analysis in the Dentate Gyrus**

**Supplementary** **Table 15. WGCNA GOanRichment Analysis in the Hippocampal CA1-3**

**Figure legends:**

**Supplementary** **Figure 1. Clustering of cases and corresponding clinical variables in each brain region.** Cases were clustered with the WGCNA R package, based on protein expression identified by LC-MS/MS on top, and on bottom are the corresponding clinical variables in a heatmap. Clinical variables and combinations can be evaluated (i.e. whether cases cluster by FS history and NP HP findings) in **a)** the frontal cortex, **b)** dentate gyrus, and **c)** hippocampal CA1-3. Similar to the PCA in Fig. 1, there is more clustering of SUDC cases in the frontal cortex.

**Supplementary** **Figure 2. Significant proteins in frontal cortex of SUDC cases negatively correlate with epilepsy.** To determine whether there were similarities in SUDC cases to epilepsy, a correlation analysis was performed in each brain region analyzed. A comparison of significant SUDC proteins and our previous epilepsy analysis [60] indicated **a)** 82/660 SUDC significant proteins were also significant in epilepsy frontal cortex, **b)** 2/171 in dentate gyrus, and **c)** 11/57 in hippocampal CA1-3. **d)** Of the 82 common proteins in frontal cortex of SUDC and epilepsy, there was a negative correlation of fold change (p < 0.0001, R^2^ = 0.39). There were 15/82 proteins that had a fold change in the same direction (purple) and 67/82 in the opposite direction (green). COX6B1 had the largest fold change in both SUDC and epilepsy, which was decreased in both groups.

**Supplementary** **Figure 1.**


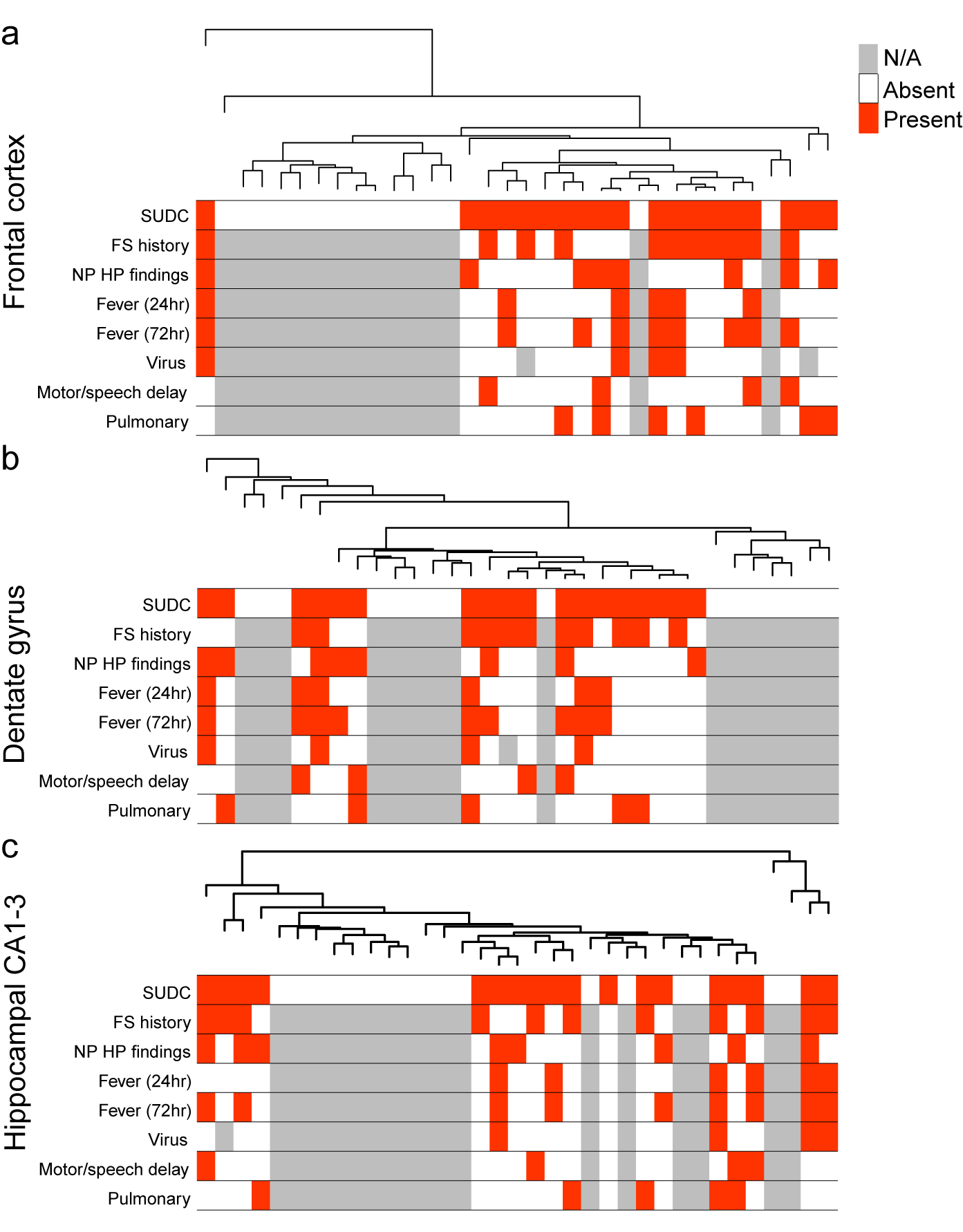


**Supplementary** **Figure 2.**


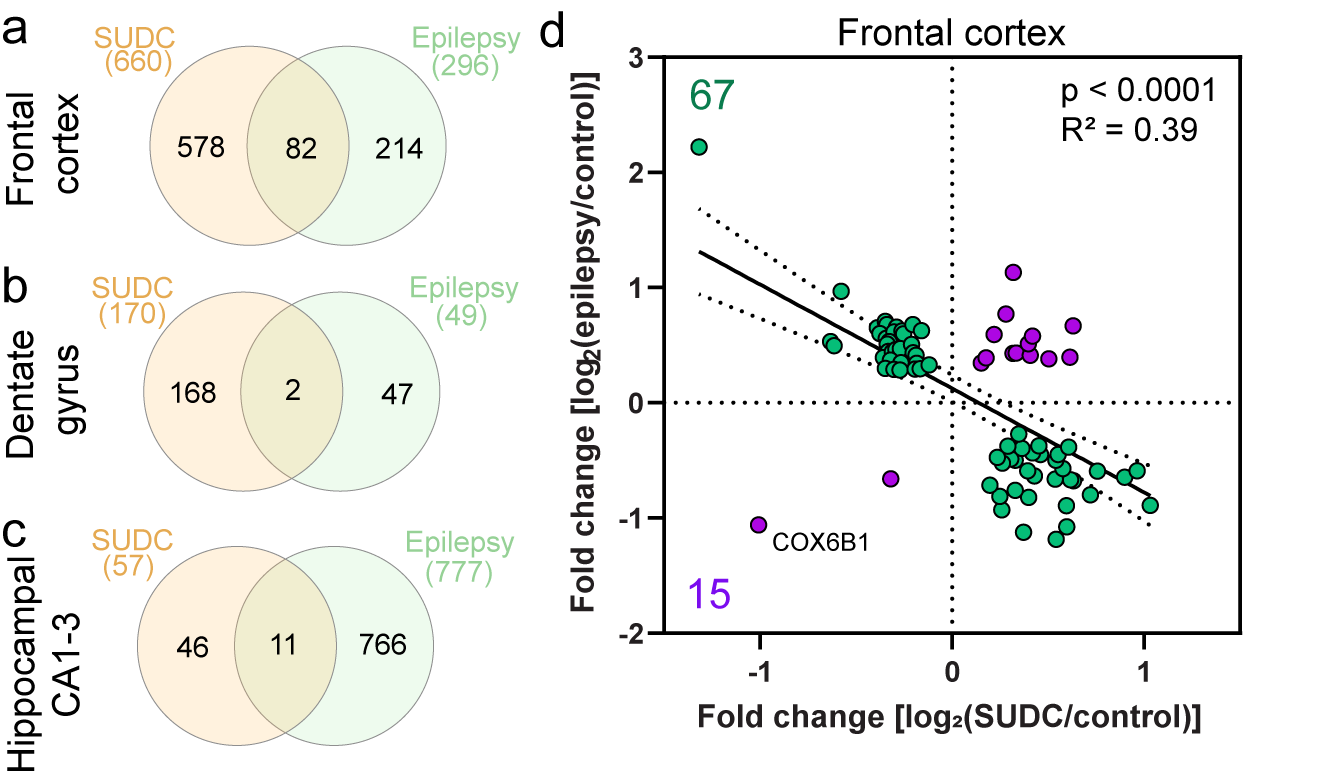

Supplement: Supplementary file 1 — Supplementary file1 (DOCX 221 kb) [file 401_2022_2414_MOESM1_ESM.docx]
